# Aqua­[1-(4-carb­oxy­phen­yl)-1*H*-imidazole-κ*N* ^3^](pyridine-2,6-dicarboxyl­ato-κ^3^ *O* ^2^,*N*,*O* ^6^)copper(II) monohydrate

**DOI:** 10.1107/S1600536811015522

**Published:** 2011-05-20

**Authors:** Fanzhen Kong, Zhangyu Yu

**Affiliations:** a School of Chemistry and Chemical Engineering, Qufu Normal University, Qufu, Shandong 330031, People’s Republic of China

## Abstract

In the title complex, [Cu(C_7_H_3_NO_4_)(C_10_H_8_N_2_O_2_)(H_2_O)]·H_2_O, the Cu^II^ ion is in a slightly distorted square-pyramidal geometry. Two carboxyl­ate O atoms and one pyridine N atom from a pyridine-2,6-dicarboxyl­ate ligand chelate the Cu^II^ ion, forming two stable five-membered metalla rings. One imidazole N atom from a 1-(4-carb­oxy­phen­yl)imidazole ligand and one water mol­ecule complete the five-coordination. O—H⋯O hydrogen bonds involving the coordinated water mol­ecules and carboxyl­ate groups link the complex mol­ecules into chain-containing dinuclear macrocycles. O—H⋯O hydrogen bonds involving the uncoordinated water mol­ecules link the chains into a layer extending parallel to (10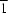).

## Related literature

For the design and synthesis of compounds with metal–organic supra­molecular architectures, see: Bradshaw *et al.* (2005 ▶); Tian *et al.* (2005 ▶); Wang *et al.* (2009 ▶). For the use of N-containing heterocyclic carboxyl­ate ligands in metal–organic supra­molecular architectures, see: Bentiss *et al.* (2004 ▶); Yang *et al.* (2008 ▶); Zeng *et al.* (2006 ▶). For related structures, see: Li *et al.* (2008 ▶).
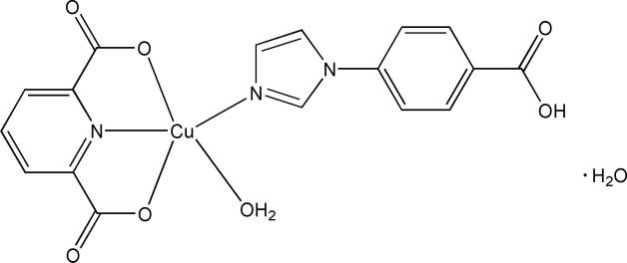


## Experimental

### 

#### Crystal data

[Cu(C_7_H_3_NO_4_)(C_10_H_8_N_2_O_2_)(H_2_O)]·H_2_O
*M* *_r_* = 452.87
Triclinic, 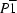

*a* = 8.1638 (5) Å
*b* = 8.2081 (5) Å
*c* = 13.1265 (18) Å
α = 84.353 (16)°
β = 85.789 (13)°
γ = 80.736 (14)°
*V* = 862.43 (15) Å^3^
*Z* = 2
Mo *K*α radiation
μ = 1.32 mm^−1^
*T* = 293 K
0.34 × 0.20 × 0.15 mm

#### Data collection

Bruker APEX CCD diffractometer
Absorption correction: multi-scan (*SADABS*; Sheldrick, 1996 ▶) *T*_min_ = 0.662, *T*_max_ = 0.826
6749 measured reflections
3920 independent reflections
3485 reflections with *I* > 2σ(*I*)
*R*_int_ = 0.020

#### Refinement

*R*[*F* ^2^ > 2σ(*F* ^2^)] = 0.036
*wR*(*F* ^2^) = 0.096
*S* = 1.06
3920 reflections
322 parameters
5 restraints
H atoms treated by a mixture of independent and constrained refinement
Δρ_max_ = 0.37 e Å^−3^
Δρ_min_ = −0.62 e Å^−3^

### 

Data collection: *SMART* (Bruker, 2007 ▶); cell refinement: *SAINT* (Bruker, 2007 ▶); data reduction: *SAINT*; program(s) used to solve structure: *SHELXS97* (Sheldrick, 2008 ▶); program(s) used to refine structure: *SHELXL97* (Sheldrick, 2008 ▶); molecular graphics: *SHELXTL* (Sheldrick, 2008 ▶); software used to prepare material for publication: *SHELXTL*.

## Supplementary Material

Crystal structure: contains datablocks I, global. DOI: 10.1107/S1600536811015522/hy2420sup1.cif

Structure factors: contains datablocks I. DOI: 10.1107/S1600536811015522/hy2420Isup2.hkl

Additional supplementary materials:  crystallographic information; 3D view; checkCIF report

## Figures and Tables

**Table 1:** Hydrogen-bond geometry (Å, °)

*D*—H⋯*A*	*D*—H	H⋯*A*	*D*⋯*A*	*D*—H⋯*A*
O1—H1⋯O3^i^	0.81 (1)	1.90 (1)	2.710 (2)	173 (4)
O1*W*—H1*W*1⋯O2*W*^ii^	0.82 (2)	2.02 (2)	2.803 (3)	159 (4)
O1*W*—H2*W*1⋯O5^ii^	0.82 (2)	1.92 (2)	2.740 (2)	177 (3)
O2*W*—H1*W*2⋯O6	0.83 (2)	1.98 (2)	2.794 (2)	168 (3)
O2*W*—H2*W*2⋯O2^iii^	0.83 (2)	2.25 (2)	2.986 (3)	148 (3)

Symmetry codes: (i) 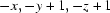; (ii) 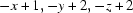; (iii) 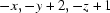.

**Fig. 1.**
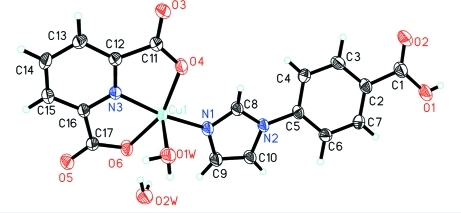
Molecular structure of the title compound. Displacement ellipsoids are drawn at the 50% probability level.

**Fig. 2.**
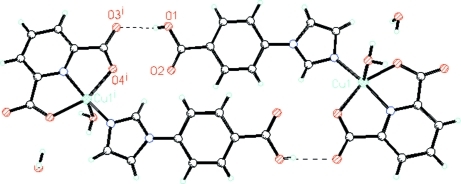
View of the dinuclear CuII supramolecular macrocycle. Dashed lines denote hydrogen bonds. [Symmetry code: (i) -x, -y+1, -z+1.]

**Fig. 3.**
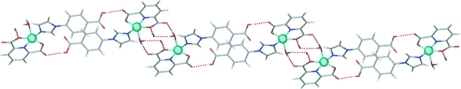
View of the one-dimensional supramolecular chain. Dashed lines denote hydrogen bonds.

## supplementary crystallographic information

### Comment

The rational design and synthesis of metal–organic supramolecular architectures are of great interest and importance owing to their intriguing structural topologies and potential applications as functional materials (Bradshaw *et al.*, 2005). A successful strategy in building the architectures is the deliberate selection of functional organic ligands and transition metal ions with specific coordination geometry. In the context, the carboxylate ligands are widely employed because they exhibit diverse coordination modes (Tian *et al.*, 2005; Wang *et al.*, 2009). The different coordination modes of carboxylate groups can induce different coordination geometries of transition metal ions and enhance the robustness of the resulting architectures. Moreover, the negative charge of carboxylate groups compensates the positive charge from metal ions and can mitigate the counterion effect in self-assembly processes. With the development of supramolecular chemistry and crystal engineering, the scope of the investigations on carboxylate ligands has been widen by the use of N-containing heterocyclic carboxylate ligands, such as pyrazole- (Bentiss *et al.*, 2004), imidazole- (Zeng *et al.*, 2006) and pyridine-carboxylates (Wang *et al.*, 2009; Yang *et al.*, 2008). The introduction of N atoms can satisfy the coordination requirements of metal ions and they also link metal–carboxylate frameworks into various fascinating extended networks. On the other hand, N atoms are highly accessible to transition metal ions, their stronger coordination ability than carboxylate groups may result in the formation of hydrogen bonding interactions by uncoordinated carboxylate O atoms, which further make the whole framework more stable. Among N–containing carboxylate ligands, we are interested in pyridine-2,6-dicarboxylic acid (2,6-H_2_pydc) since its N atom and two carboxylate groups may chelate one metal ion, forming two stable five-membered rings (Li *et al.*, 2008). The introduction of the other N-containing carboxylate ligands may lead to interesting structural frameworks. Herein, we report a Cu(II) supramolecular complex from 2,6-H_2_pydc and 4-(imidazol-1-yl)benzoic acid (HIBA).

As shown in Fig. 1, the Cu^II^ ion has a slightly distorted square-pyramidal coordination geometry. Two carboxylate O atoms and one pyridine N atom from a 2,6-pydc ligand and one imidazolyl N atom from HIBA are in the basal plane, with a mean deviation of 0.0222 (2) Å from the plane. Cu1 atom is slightly out of the plane about 0.2221 (3) Å. One water molecule (O1W) occupies the apical position. As expected, two O atoms (O4, O6) from different carboxylate groups and an N atom (N3) chelate Cu1, forming two stable five-membered rings. This results in Cu1—N3 bond distance of 1.9075 (17) Å being much shorter than Cu1—N1 bond distance of 1.9467 (18) Å. Two carboxylate groups (O3, O4, C11 and O5, O6, C17) are almost coplanar with the pyridine ring, with dihedral angles between them of 3.5 (1) and 7.3 (2)°, respectively. HIBA serves as a monodentate ligand through imidazolyl N atom coordinating to Cu1 atom. The twisting angle between the imidazolyl ring and benzene ring is 23.02 (8)°, while the dihedral angle between the benzene ring and the carboxylate group in HIBA is 2.7 (2)°.

Interestingly, carboxylic proton forms a strong hydrogen bond with one uncoordinated carboxylate O atom of the 2,6-pydc ligand (Table 1), which results in a dinuclear supramolecular macrocycle (Fig. 2). The Cu···Cu separation in the cycle is 13.749 (2) Å. O—H···O hydrogen bonds between the other carboxylate group of the 2,6-pydc ligand and the coordinated water molecule link the macrocyles into a one-dimensional supramolecular chain (Fig. 3). The nearest Cu···Cu separation in the chain is 6.322 (1) Å. O—H···O hydrogen bonds involving the uncoordinated water molecules link the chains into a layer.

### Experimental

A mixture of pyridine-2,6-dicarboxylic acid (0.034 g, 0.2 mmol), HIBA (0.038 g, 0.2 mmol), copper nitrate hydrate (0.036 g, 0.2 mmol) and one drop of KOH aqueous solution (10%) in 15 ml distilled water was heated in a 30 ml Teflon-lined steel bomb at 433 K for 3 d. Blue crystals formed were collected, washed with ethanol and dried in air.

### Refinement

All H atoms were located from difference Fourier maps and refined isotropically, with a distance restraint of O—H = 0.82 (1) Å.

### Crystal data

**Table:**

[Cu(C_7_H_3_NO_4_)(C_10_H_8_N_2_O_2_)(H_2_O)]·H_2_O	*Z* = 2
*M**_r_* = 452.87	*F*(000) = 462
Triclinic, *P*1	*D*_x_ = 1.744 Mg m^−^^3^
Hall symbol: -P 1	Mo *K*α radiation, λ = 0.71073 Å
*a* = 8.1638 (5) Å	Cell parameters from 6749 reflections
*b* = 8.2081 (5) Å	θ = 2.9–27.5°
*c* = 13.1265 (18) Å	µ = 1.32 mm^−^^1^
α = 84.353 (16)°	*T* = 293 K
β = 85.789 (13)°	Block, blue
γ = 80.736 (14)°	0.34 × 0.20 × 0.15 mm
*V* = 862.43 (15) Å^3^	

### Data collection

**Table:**

Bruker APEX CCD diffractometer	3920 independent reflections
Radiation source: fine-focus sealed tube	3485 reflections with *I* > 2σ(*I*)
graphite	*R*_int_ = 0.020
φ and ω scans	θ_max_ = 27.5°, θ_min_ = 2.9°
Absorption correction: multi-scan (*SADABS*; Sheldrick, 1996)	*h* = −10→10
*T*_min_ = 0.662, *T*_max_ = 0.826	*k* = −10→10
6749 measured reflections	*l* = −14→17

### Refinement

**Table:**

Refinement on *F*^2^	Primary atom site location: structure-invariant direct methods
Least-squares matrix: full	Secondary atom site location: difference Fourier map
*R*[*F*^2^ > 2σ(*F*^2^)] = 0.036	Hydrogen site location: inferred from neighbouring sites
*wR*(*F*^2^) = 0.096	H atoms treated by a mixture of independent and constrained refinement
*S* = 1.06	*w* = 1/[σ^2^(*F*_o_^2^) + (0.0513*P*)^2^ + 0.3344*P*] where *P* = (*F*_o_^2^ + 2*F*_c_^2^)/3
3920 reflections	(Δ/σ)_max_ = 0.001
322 parameters	Δρ_max_ = 0.37 e Å^−^^3^
5 restraints	Δρ_min_ = −0.62 e Å^−^^3^

### Fractional atomic coordinates and isotropic or equivalent isotropic displacement parameters (Å^2^)

**Table:**

	*x*	*y*	*z*	*U*_iso_*/*U*_eq_	
Cu1	0.51191 (3)	0.77853 (3)	0.81495 (2)	0.02775 (11)	
O1	−0.5172 (2)	0.7396 (3)	0.42103 (17)	0.0516 (5)	
N1	0.3000 (2)	0.8750 (2)	0.75900 (14)	0.0286 (4)	
C1	−0.3561 (3)	0.6898 (3)	0.40439 (19)	0.0347 (5)	
H1	−0.575 (4)	0.708 (5)	0.381 (2)	0.076 (12)*	
O2	−0.2974 (2)	0.6052 (3)	0.33628 (16)	0.0533 (5)	
N2	0.0788 (2)	0.8924 (2)	0.67065 (13)	0.0245 (4)	
C2	−0.2492 (3)	0.7482 (3)	0.47641 (17)	0.0283 (5)	
O3	0.7305 (2)	0.3533 (2)	0.70285 (15)	0.0414 (4)	
N3	0.7383 (2)	0.6954 (2)	0.84101 (14)	0.0244 (4)	
C3	−0.0783 (3)	0.6968 (3)	0.46374 (18)	0.0312 (5)	
H3	−0.036 (4)	0.627 (4)	0.408 (2)	0.046 (8)*	
O4	0.5460 (2)	0.5751 (2)	0.73733 (14)	0.0376 (4)	
C4	0.0295 (3)	0.7460 (3)	0.52654 (18)	0.0300 (5)	
H4	0.135 (4)	0.714 (3)	0.515 (2)	0.038 (7)*	
O5	0.7779 (2)	1.0466 (2)	0.96052 (14)	0.0392 (4)	
C5	−0.0332 (3)	0.8478 (2)	0.60358 (16)	0.0237 (4)	
O6	0.57541 (19)	0.97815 (19)	0.87486 (13)	0.0333 (4)	
C6	−0.2030 (3)	0.9038 (3)	0.61558 (17)	0.0290 (4)	
H6	−0.247 (3)	0.981 (3)	0.673 (2)	0.039 (7)*	
C7	−0.3103 (3)	0.8539 (3)	0.55223 (18)	0.0305 (5)	
H7	−0.418 (4)	0.888 (3)	0.562 (2)	0.036 (7)*	
C8	0.2294 (3)	0.8052 (3)	0.69116 (17)	0.0282 (4)	
H8	0.265 (3)	0.706 (3)	0.6699 (18)	0.026 (6)*	
C9	0.1899 (3)	1.0144 (3)	0.78355 (18)	0.0290 (5)	
H9	0.213 (3)	1.084 (3)	0.830 (2)	0.030 (6)*	
C10	0.0526 (3)	1.0255 (3)	0.72996 (17)	0.0280 (4)	
H10	−0.043 (3)	1.102 (3)	0.726 (2)	0.033 (7)*	
C11	0.6874 (3)	0.4845 (3)	0.74368 (17)	0.0291 (5)	
C12	0.8070 (3)	0.5506 (3)	0.80631 (17)	0.0255 (4)	
C13	0.9705 (3)	0.4866 (3)	0.82600 (19)	0.0309 (5)	
H13	1.023 (4)	0.383 (4)	0.803 (2)	0.052 (8)*	
C14	1.0569 (3)	0.5759 (3)	0.88262 (19)	0.0324 (5)	
H14	1.165 (4)	0.537 (4)	0.895 (2)	0.049 (8)*	
C15	0.9826 (3)	0.7273 (3)	0.91638 (18)	0.0287 (4)	
H15	1.038 (3)	0.791 (3)	0.955 (2)	0.037 (7)*	
C16	0.8205 (3)	0.7846 (3)	0.89290 (16)	0.0240 (4)	
C17	0.7182 (3)	0.9512 (3)	0.91352 (17)	0.0269 (4)	
O1W	0.4032 (2)	0.6744 (2)	0.96552 (14)	0.0357 (4)	
O2W	0.3880 (2)	1.2940 (2)	0.87970 (16)	0.0427 (4)	
H1W1	0.474 (4)	0.658 (5)	1.008 (2)	0.074 (12)*	
H2W1	0.349 (4)	0.756 (3)	0.990 (2)	0.055 (9)*	
H1W2	0.445 (4)	1.203 (3)	0.869 (2)	0.054 (9)*	
H2W2	0.396 (4)	1.341 (4)	0.8208 (17)	0.061 (11)*	

### Atomic displacement parameters (Å^2^)

**Table:**

	*U*^11^	*U*^22^	*U*^33^	*U*^12^	*U*^13^	*U*^23^
Cu1	0.02014 (15)	0.02746 (16)	0.03696 (18)	0.00146 (10)	−0.01098 (11)	−0.01095 (11)
O1	0.0321 (10)	0.0655 (13)	0.0633 (13)	0.0001 (9)	−0.0194 (9)	−0.0361 (11)
N1	0.0232 (9)	0.0299 (9)	0.0334 (10)	0.0004 (7)	−0.0105 (7)	−0.0080 (7)
C1	0.0339 (12)	0.0319 (12)	0.0404 (13)	−0.0037 (9)	−0.0120 (10)	−0.0084 (10)
O2	0.0403 (10)	0.0672 (13)	0.0592 (13)	−0.0047 (9)	−0.0134 (9)	−0.0379 (11)
N2	0.0224 (8)	0.0254 (9)	0.0266 (9)	−0.0013 (7)	−0.0083 (7)	−0.0049 (7)
C2	0.0292 (11)	0.0288 (11)	0.0283 (11)	−0.0037 (9)	−0.0113 (9)	−0.0029 (8)
O3	0.0322 (9)	0.0394 (9)	0.0561 (11)	0.0020 (7)	−0.0108 (8)	−0.0277 (8)
N3	0.0204 (8)	0.0244 (8)	0.0293 (9)	−0.0010 (7)	−0.0051 (7)	−0.0077 (7)
C3	0.0335 (12)	0.0317 (11)	0.0290 (11)	−0.0005 (9)	−0.0074 (9)	−0.0092 (9)
O4	0.0268 (8)	0.0371 (9)	0.0522 (10)	0.0015 (7)	−0.0155 (7)	−0.0211 (8)
C4	0.0224 (10)	0.0351 (12)	0.0325 (11)	0.0003 (9)	−0.0063 (9)	−0.0073 (9)
O5	0.0319 (9)	0.0365 (9)	0.0534 (11)	−0.0019 (7)	−0.0107 (8)	−0.0240 (8)
C5	0.0238 (10)	0.0247 (10)	0.0232 (10)	−0.0031 (8)	−0.0087 (8)	−0.0012 (8)
O6	0.0265 (8)	0.0262 (8)	0.0488 (10)	0.0031 (6)	−0.0151 (7)	−0.0131 (7)
C6	0.0258 (10)	0.0341 (11)	0.0273 (11)	0.0003 (9)	−0.0065 (8)	−0.0074 (9)
C7	0.0214 (10)	0.0391 (12)	0.0318 (11)	−0.0018 (9)	−0.0070 (9)	−0.0074 (9)
C8	0.0238 (10)	0.0287 (11)	0.0326 (11)	0.0021 (8)	−0.0104 (8)	−0.0084 (9)
C9	0.0278 (11)	0.0257 (10)	0.0340 (12)	0.0001 (8)	−0.0097 (9)	−0.0073 (9)
C10	0.0264 (11)	0.0259 (10)	0.0321 (11)	0.0006 (9)	−0.0083 (9)	−0.0075 (8)
C11	0.0248 (10)	0.0319 (11)	0.0327 (11)	−0.0037 (9)	−0.0055 (9)	−0.0120 (9)
C12	0.0224 (10)	0.0255 (10)	0.0294 (11)	−0.0021 (8)	−0.0038 (8)	−0.0077 (8)
C13	0.0244 (11)	0.0280 (11)	0.0397 (13)	0.0038 (9)	−0.0053 (9)	−0.0097 (9)
C14	0.0202 (10)	0.0355 (12)	0.0413 (13)	0.0017 (9)	−0.0085 (9)	−0.0071 (10)
C15	0.0250 (10)	0.0328 (11)	0.0303 (11)	−0.0055 (9)	−0.0074 (9)	−0.0068 (9)
C16	0.0216 (9)	0.0242 (10)	0.0272 (10)	−0.0030 (8)	−0.0050 (8)	−0.0060 (8)
C17	0.0229 (10)	0.0274 (10)	0.0314 (11)	−0.0019 (8)	−0.0043 (8)	−0.0085 (8)
O1W	0.0334 (9)	0.0336 (9)	0.0407 (10)	0.0007 (7)	−0.0077 (8)	−0.0119 (7)
O2W	0.0458 (11)	0.0343 (10)	0.0467 (11)	0.0060 (8)	−0.0110 (9)	−0.0103 (8)

### Geometric parameters (Å, °)

**Table:**

Cu1—N1	1.9467 (18)	O5—C17	1.222 (3)
Cu1—N3	1.9076 (17)	C5—C6	1.391 (3)
Cu1—O4	2.0118 (16)	O6—C17	1.283 (3)
Cu1—O6	2.0393 (16)	C6—C7	1.385 (3)
Cu1—O1W	2.2537 (19)	C6—H6	1.04 (3)
O1—C1	1.322 (3)	C7—H7	0.88 (3)
O1—H1	0.81 (1)	C8—H8	0.89 (3)
N1—C8	1.318 (3)	C9—C10	1.353 (3)
N1—C9	1.386 (3)	C9—H9	0.92 (3)
C1—O2	1.210 (3)	C10—H10	0.92 (3)
C1—C2	1.493 (3)	C11—C12	1.517 (3)
N2—C8	1.350 (3)	C12—C13	1.386 (3)
N2—C10	1.383 (3)	C13—C14	1.392 (3)
N2—C5	1.426 (3)	C13—H13	0.95 (3)
C2—C3	1.393 (3)	C14—C15	1.391 (3)
C2—C7	1.396 (3)	C14—H14	0.91 (3)
O3—C11	1.239 (3)	C15—C16	1.378 (3)
N3—C16	1.332 (3)	C15—H15	0.95 (3)
N3—C12	1.337 (3)	C16—C17	1.521 (3)
C3—C4	1.379 (3)	O1W—H1W1	0.82 (2)
C3—H3	0.98 (3)	O1W—H2W1	0.82 (2)
O4—C11	1.272 (3)	O2W—H1W2	0.83 (2)
C4—C5	1.391 (3)	O2W—H2W2	0.83 (2)
C4—H4	0.87 (3)		
			
N3—Cu1—N1	167.81 (8)	C5—C6—H6	119.4 (16)
N3—Cu1—O4	80.11 (7)	C6—C7—C2	120.5 (2)
N1—Cu1—O4	95.85 (7)	C6—C7—H7	118.2 (18)
N3—Cu1—O6	80.22 (7)	C2—C7—H7	121.3 (18)
N1—Cu1—O6	100.91 (7)	N1—C8—N2	110.75 (19)
O4—Cu1—O6	156.95 (7)	N1—C8—H8	125.3 (16)
N3—Cu1—O1W	96.05 (7)	N2—C8—H8	123.1 (16)
N1—Cu1—O1W	95.97 (8)	C10—C9—N1	109.0 (2)
O4—Cu1—O1W	99.71 (7)	C10—C9—H9	128.9 (16)
O6—Cu1—O1W	94.19 (7)	N1—C9—H9	122.2 (16)
C1—O1—H1	114 (3)	C9—C10—N2	106.46 (19)
C8—N1—C9	106.55 (18)	C9—C10—H10	132.6 (17)
C8—N1—Cu1	123.09 (15)	N2—C10—H10	120.9 (17)
C9—N1—Cu1	130.21 (15)	O3—C11—O4	125.2 (2)
O2—C1—O1	123.7 (2)	O3—C11—C12	120.44 (19)
O2—C1—C2	121.8 (2)	O4—C11—C12	114.40 (18)
O1—C1—C2	114.5 (2)	N3—C12—C13	119.5 (2)
C8—N2—C10	107.26 (18)	N3—C12—C11	111.02 (18)
C8—N2—C5	125.26 (18)	C13—C12—C11	129.41 (19)
C10—N2—C5	127.41 (18)	C12—C13—C14	118.2 (2)
C3—C2—C7	119.1 (2)	C12—C13—H13	122 (2)
C3—C2—C1	117.1 (2)	C14—C13—H13	119.8 (19)
C7—C2—C1	123.8 (2)	C15—C14—C13	120.9 (2)
C16—N3—C12	123.10 (18)	C15—C14—H14	119.7 (19)
C16—N3—Cu1	118.33 (14)	C13—C14—H14	119.3 (19)
C12—N3—Cu1	118.58 (15)	C16—C15—C14	117.7 (2)
C4—C3—C2	120.8 (2)	C16—C15—H15	119.2 (17)
C4—C3—H3	120.8 (18)	C14—C15—H15	123.0 (17)
C2—C3—H3	118.4 (18)	N3—C16—C15	120.51 (19)
C11—O4—Cu1	115.70 (14)	N3—C16—C17	111.96 (18)
C3—C4—C5	119.6 (2)	C15—C16—C17	127.41 (19)
C3—C4—H4	117.9 (19)	O5—C17—O6	126.6 (2)
C5—C4—H4	122.4 (19)	O5—C17—C16	119.27 (19)
C6—C5—C4	120.4 (2)	O6—C17—C16	114.11 (18)
C6—C5—N2	120.54 (18)	Cu1—O1W—H1W1	110 (3)
C4—C5—N2	119.10 (19)	Cu1—O1W—H2W1	104 (2)
C17—O6—Cu1	114.41 (13)	H1W1—O1W—H2W1	96 (3)
C7—C6—C5	119.6 (2)	H1W2—O2W—H2W2	98 (3)
C7—C6—H6	121.0 (16)		

### Hydrogen-bond geometry (Å, °)

**Table:**

*D*—H···*A*	*D*—H	H···*A*	*D*···*A*	*D*—H···*A*
O1—H1···O3^i^	0.81 (1)	1.90 (1)	2.710 (2)	173 (4)
O1W—H1W1···O2W^ii^	0.82 (2)	2.02 (2)	2.803 (3)	159 (4)
O1W—H2W1···O5^ii^	0.82 (2)	1.92 (2)	2.740 (2)	177 (3)
O2W—H1W2···O6	0.83 (2)	1.98 (2)	2.794 (2)	168 (3)
O2W—H2W2···O2^iii^	0.83 (2)	2.25 (2)	2.986 (3)	148 (3)

Symmetry codes: (i) −*x*, −*y*+1, −*z*+1; (ii) −*x*+1, −*y*+2, −*z*+2; (iii) −*x*, −*y*+2, −*z*+1.
